# Valorization of khat (*Catha edulis*) waste for the production of cellulose fibers and nanocrystals

**DOI:** 10.1371/journal.pone.0246794

**Published:** 2021-02-09

**Authors:** Tesfaye Gabriel, Kebede Wondu, Jemal Dilebo

**Affiliations:** Department of Pharmaceutics and Social Pharmacy, School of Pharmacy, College of Health Sciences, Addis Ababa University, Addis Ababa, Ethiopia; University of Naples Federico II, ITALY

## Abstract

Cellulose fibers (C_40_ and C_80_) were extracted from khat (*Catha edulis*) waste (KW) with chlorine-free process using 40% formic acid/40% acetic acid (C_40_), and 80% formic acid/80% acetic acid (C_80_) at the pretreatment stage, followed by further delignification and bleaching stages. Cellulose nanocrystals (CNCs_40_ and CNCs_80_) were then isolated from C_40_ and C_80_ with sulfuric acid hydrolysis, respectively. Thus, the current study aims to isolate cellulose fibers and CNCs from KW as alternative source. The KW, cellulose fibers, and CNCs were investigated for yield, chemical composition, functionality, crystallinity, morphology, and thermal stability. CNCs were also evaluated for colloidal stability, particle size, and their influence on *in vitro* diclofenac sodium release from gel formulations preliminarily. The FTIR spectra analysis showed the removal of most hemicellulose and lignin from the cellulose fibers. The XRD results indicated that chemical pretreatments and acid hydrolysis significantly increased the crystallinity of cellulose fibers and CNCs. The cellulose fibers and CNCs exhibited Cellulose I_β_ crystalline lattice. TEM analysis revealed formation of needle-shaped nanoscale rods (length: 101.55–162.96 nm; aspect ratio: 12.84–22.73). The hydrodynamic size, polydispersity index, and zeta potential of the CNC_S_ ranged from 222.8–362.8 nm; 0.297–0.461, and -45.7 to -75.3 mV, respectively. CNCs_40_ exhibited superior properties to CNCs_80_ in terms of aspect ratio, and colloidal and thermal stability. Gel formulations containing high proportion of CNCs sustained diclofenac sodium release (< 50%/cm^2^) over 12 h. This study suggests that cellulose fibers and nanocrystals can be successfully obtained from abundant and unexploited source, KW for value-added industrial applications.

## Introduction

Khat or chat (*Catha edulis* Forsk) is an evergreen shrub native to the Horn of Africa and the Middle East. Its fresh leaves are chewed by millions worldwide as a recreational drug on daily bases for its euphoric and psychostimulant effect [1]. Ethiopia is the world’s largest producer of khat which has recently become the fastest growing export commodity [2, 3]. Khat has been used for generations by all walks of life, including children, pregnant, breastfeeding women and patients on medication [4]. Due to economic attractiveness, khat cultivation by local farmers is expanding with rapid reduction of annual crops production though the Ethiopian law on the issue of khat is in limbo neither supporting nor denouncing its use [5]. Over two million farmers produce khat on more than 250,000 ha of land [6].

When the young leaves of khat are collected for chewing locally and export, most parts of the plant such as older leaves and twigs are dumped as a solid waste. The large quantities of khat solid waste in the cities and towns have reduced the beauty of the cities/towns and became a breeding place of some rodents and vectors. Moreover, the waste encourages some people to dispose more and therefore exacerbates the poor sanitation in the cities and towns. As the waste production is so enormous, it needs proper disposal mechanism [3]. The conversion of such a waste material into useful products such as cellulose and its derivatives would alleviate a variety of socioeconomic problems, providing a greener approach to utilizing the waste through addressing the ecological and economic issues: preventing deforestation and environmental pollution, and generating foreign currency.

Recently, renewable natural resources for the development of recyclable and/or biodegradable products have received much attention to protect the environment from pollution. Lignocellulosic materials are among the most important natural sources for the production of value-added materials or biopolymers [7]. Cellulose is the most abundant polymer in nature which can be obtained from plants, animals, or bacteria. Cellulose is preferred to other polymers because of its biodegradability, abundance, light weight, cost effectiveness, high tensile strength and stiffness [8]. Cellulose and its derivatives are commonly obtained from woody plants and cotton for different industrial applications. The overuse of such sources for years by various industries such as energy and construction, has increased the need for alternative cellulose sources [9].

Nanocelluloses, also known as cellulose nanomaterials, have attracted rapidly growing scientific and technological interest from both academic and industrial researchers [10]. The two main classes of nanocelluloses are a) cellulose nanocrystals (CNCs), also referred to as nanocrystalline cellulose and cellulose nanowhiskers, and b) cellulose nanofibrils, also referred to as nano-fibrillated cellulose [11–13]. Due to higher surface area, reactive OH group in the surface, and biocompatibility among other properties, CNCs are suitable for many advanced functional applications such as tissue engineering, drug delivery, reinforcement of composite materials, etc [11, 14].

Recently, various studies have reported work of waste valorization for the production of nanocellulose from different lignocellulosic sources such as pineapple peel waste [15], *Posidonia oceanica* waste [16–18], garlic straw residues [19], industrial kelp waste [20], grass waste [21], spent coffee grounds [22], paper mill sludge [23], paper waste [24], etc.

To the best of the author’s knowledge, there is no report on isolation and characterization of cellulose fibers and CNCs from an unexploited and abundant source, khat waste (KW) for potential value-added applications. The raw KW, as-extracted cellulose fibers and as-isolated CNCs were characterized with Fourier transform infrared spectroscopy (FTIR), X-ray diffraction (XRD), Scanning Electron Microscopy (SEM), Transmission Electron Microscopy (TEM), Dynamic Light Scattering (DLS), and Thermogravimetric Analysis (TGA). The CNCs were also evaluated preliminarily as a reinforcing material in carboxymethyl cellulose gel for controlled delivery of diclofenac sodium topically. CNCs offer several potential advantages in the drug delivery system. Large amounts of drugs might be bound to the surface of CNCs with the potential for high payloads and optimal control of dosing due to large surface area and negative charge [25]. Recently, CNCs-chitosan based hydrogel was fabricated for controlled delivery of theophylline, and the interaction between CNCs and chitosan is due to H-bond, van der Waals forces, and ionic and/or covalent bonds [26]. It was also reported that drugs (such as doxorubicin hydrochloride and tetracycline hydrochloride) carrying a positive charge under physiological pH conditions, probably form strong ionic bonds with the negatively charged sulfate groups resident on the CNC surface as a result of the sulfuric acid hydrolysis process [27]. Diclofenac sodium is a non-steroidal anti-inflammatory drug widely used in the management of different inflammatory conditions, but it has short half-life around 2 h [28]. Topical/transdermal drug delivery is an attractive alternative to conventional methods because of advantages such as non-invasive delivery, constant and steady levels of drug with short biological half-life, no first-pass effect, prolonged duration of action, reduced dosing frequency, reduced drug toxicity/adverse effects, and improved patient compliance among others [29]. Thus, the aim of this study was to valorize the KW as a new and alternative source for production of cellulose and CNCs using eco-friendly method for controlled drug delivery.

## Materials and methods

### Materials

Khat waste was collected from Obsa Special Khat Shop, Addis Ababa, Ethiopia and cut into small pieces. Formic acid (98%) (Central Drug House (P) Ltd. New Delhi, India), acetone and sodium hydroxide 97% (HiMedia, Mumbai, India), acetic acid 99.8% (Riedel-de Haën), sulfuric acid 97% (BDH, England), copper sulfate pentahydrate 98.5%, n-hexane 99% and zinc chloride (LOBA CHEMIE-Laboratory, India), toluene (Fisher Scientific, UK), potassium iodide (Reagent chemical services Ltd Runcorn, Cheshire), ammonium oxalate 99.5% (UNI-Chem, Chemical Reagents), iodine 99%, methanol 99.9%, and ammonia solution 28% (CARLO ERBA reagents, France), ethanol absolute (Fisher Scientific, UK), hydrogen peroxide 50% (Awash Melkasa, Ethiopia), diclofenac sodium (Healthcare Limited PLC, India), triethanolamine (Fischer Chem Alert Guide, USA), potassium dihydrogen phosphate (Sörensen, Leuren, Denmark), disodium hydrogen phosphate (Fizmerk chemicals, India), sodium chloride (Oxford Laboratory, Mumbai, India), sodium carboxymethylcellulose (FMC Corporation, USA), and propylene glycol (Research-lab fine Chem. Industries, India) were used as received.

### Cellulose extraction

Cellulose fibers were extracted from KW following chlorine-free conditions according to our previous method [9] with some modifications. Briefly, KW (100 g) was treated with formic acid/acetic acid (40%/40% or 80%/80% w/v) at a ratio of 70:30 of the two acids, and a waste to liquor ratio of 1:10 at the pretreatment stage on a water bath at 90°C for 1.5 h. After repeated washing and filtration, the pulps were then treated with 2.5% NaOH for 1 h, followed with a mixture of 20% formic acid/20% acetic acid/7.5% hydrogen peroxide (2:1:2) solution on a water bath at 90°C for 1.5 h, at a waste to liquor ratio of 1:10 with continuous washing with hot water. Lastly, bleaching was performed using 10% hydrogen peroxide in alkaline media (adding 40 g of sodium hydroxide) at 1:10 fiber ratio, first at room temperature, followed by heating on water bath at 70°C, for 30 min each. The pulps were then washed continually with hot distilled water, and finally dried in an oven (Kottermann^®^ 2711, Germany) for 1 day at 60°C. The as-obtained cellulose fibers were named as C_40_ and C_80_, respectively.

### Isolation of CNCs

CNCs were isolated from KW following the method described elsewhere with few modifications [7, 30–33]. The cellulose fibers (C_40_ and C_80_) extracted from KW were first hydrolyzed with 64% (w/w) sulfuric acid (1:20 g/mL) at 45°C for 60 min under magnetic stirrer (the resulting NCs designated as CNCs_40_ and CNCs_80_). The mixture was then diluted 10-fold with ice cubes to stop the reaction, and washed by successive centrifugations at 4°C (Beckman Coulter Allegra 64R Refrigerated Centrifuge, USA) for 10 min each at 10,000 rpm. The mixture was also dialyzed against distilled water using dialysis sacks (Avg. flat width 35 mm, MWCO 12,000 Da, Sigma-Aldrich, USA) until neutral pH was reached (5 days). Subsequently, the resulting suspension was homogenized using a disperser type UltraTurrax (Janke and Kunkel IKA-Labortechnik, Ultra-Turrax T50) for 5 min at 10,000 rpm twice and sonicated (Sonics and Materials Inc. Vibracell, VCX 750, Newtown CT, USA) in an ice bath for 5 min. The aqueous suspension was freeze-dried in a lyophilizer (Operon Co., Ltd.—Bio-Equip, Korea) and dried for 72 h to obtain CNCs powder. The yields of CNCs were estimated gravimetrically considering the initial weight of the extracted cellulose fibers (C_40_ and C_80_).

### Composition of the untreated KW and cellulose, and identification tests

The constituents of the untreated KW as well as as-obtained cellulose fibers such as lignin, hemicellulose and others were determined according to the methods stated elsewhere [9, 34–37], as described in the S1 Appendix. Solubility, appearance, color test, pH, ash values and moisture content of the as-obtained cellulose fibers from KW were determined according to the methods described in pharmacopeia [38].

### Characterization of the materials

#### Fourier-transform infrared (FTIR) spectroscopy

FTIR tests were performed on a Perkin Elmer FTIR spectrometer (L1600400 Spectrum TWO DTGS, SN: 108152, LIantrisant, UK) in the infrared range from 4000 to 450 cm^-1^, with no further sample preparation.

#### X-ray diffraction (XRD)

XRD was performed to investigate the crystallinity of as-isolated CNCs and cellulose precursors using an XRD-7000 X-ray Diffractometer MAXima (SHIMADZU Corporation, Japan) at 40 kV, 30 mA with monochromatic Cu-Kα radiation. XRD data were collected over an angular range of 10 to 40° in a sampling pitch of 0.0200° and scan speed of 3.0000°/min at room temperature.

The crystalline index (CrI) was determined using the equation proposed by Segal et al. (1959) (Empirical method):

$$CrI=\frac{{(I}_{200}-I_{am})}{I_{200}}x100\%$$

Where, *I*_*200*_ is the maximum intensity (in arbitrary units) of the diffraction from the 200 plane, and *I*_*am*_ is the intensity of the background scatter.

X-ray diffraction of the cellulose fibers and as-isolated CNCs were deconvoluted following Gaussian profile, and parameters such as d-spacings (d), apparent crystallite size or thickness for the 200 plane (τ_200_), fractional variation in the plane spacing for the 200 plane (Δd/d)_200_, the proportion of crystallite interior chains for the 200 plane (X_200_), and Z-values were determined using equations described elsewhere [8, 9, 39, 40], and provided in the S2 Appendix.

#### Environmental Scanning Electron Microscopy (ESEM)

ESEM observations were evaluated using ESEM FEI/Philips XL-30 ESEM (Leuven, Belgium) at accelerating voltage of 2.00 KV. The samples were coated with chromium using vacuum sputter prior to SEM analysis.

#### Transmission Electron Microscopy (TEM)

Droplets of CNCs suspension (0.05% w/v) were deposited on a formvar-coated copper grid. The specimen was negatively stained with a 1% (w/v) phosphotungstic acid solution and dried at room temperature. The TEM observations were conducted using an EM 900 TEM (Carl Zeiss Microscopy, Jena, Germany; acceleration voltage 80 kV), and the micrographs were taken with a slow scan camera (Variospeed SSCCD camera SM-1k-120, TRS, Moorenweis, Germany).

#### Particle size analysis

Particle size and PDI analysis of the CNCs were determined by photon correlation spectroscopy (PCS) using a 90Plus Particle Size Analyzer 28 (Brookhaven Instruments Corporation, New York, USA). Prior to the measurements, the CNCs suspensions were diluted using distilled water to yield an appropriate scattering intensity. All experiments were done at least in triplicate at 25°C.

#### Zeta Potential (ZP)

The ZP of aqueous suspension of CNCs (0.05% w/v) in 0.1N PBS was measured using Malvern Instruments Zetasizer Nano ZS working on electrophoretic mobility at 25°C after 120 s equilibration time and a wavelength of 659 nm.

#### Thermal analysis

The thermogravimetric analysis (TGA) and its derivative (DTG) of the as-obtained CNCs and cellulose precursors were studied TGA/DTG-60H (SHIMADZU Corporation, Japan). The samples were heated from room temperature to 700°C at a heating rate of 10°C/min under a nitrogen gas flow rate of 60 mL/min.

### Preparation of diclofenac sodium gel formulations

CNCs colloidal dispersions (0.25 to 2%) and CMC at a concentration of 2% (w/w) were prepared as gelling agent. Five of the medicated formulations (F1-F5) were prepared varying the concentrations of CNCs according to the formulae given in Table 1, and the other formulation containing no CNC. The medicated hydroalcoholic gel formulations were prepared by dissolving diclofenac sodium and sodium benzoate (SB) in a co-solvent of ethanol and propylene glycol (PG). Subsequently, the solution containing the drug was added to the gelling agent prepared in a known portion of distilled water under continuous stirring to yield a homogenous dispersion, which was in turn neutralized with triethanolamine to obtain a colorless gel. The final weight of the formulations was finally adjusted with distilled water [41–43].

**Table 1:** Composition % (w/w) of different diclofenac sodium gel formulations.

Formulation	Diclofenac sodium (g)	CMC (g)	CNCs (g)	Ethanol (g)	PG (g)	Triethanolamine (g)	SB (g)	Distilled water qs (g)
**F0**	3	2	0	10	10	3	0.135	100
**F1**	3	2	0.25	10	10	3	0.135	100
**F2**	3	2	0.5	10	10	3	0.135	100
**F3**	3	2	0.75	10	10	3	0.135	100
**F4**	3	2	1	10	10	3	0.135	100
**F5**	3	2	2	10	10	3	0.135	100

### UV calibration curve of diclofenac sodium

**Fig 1:**
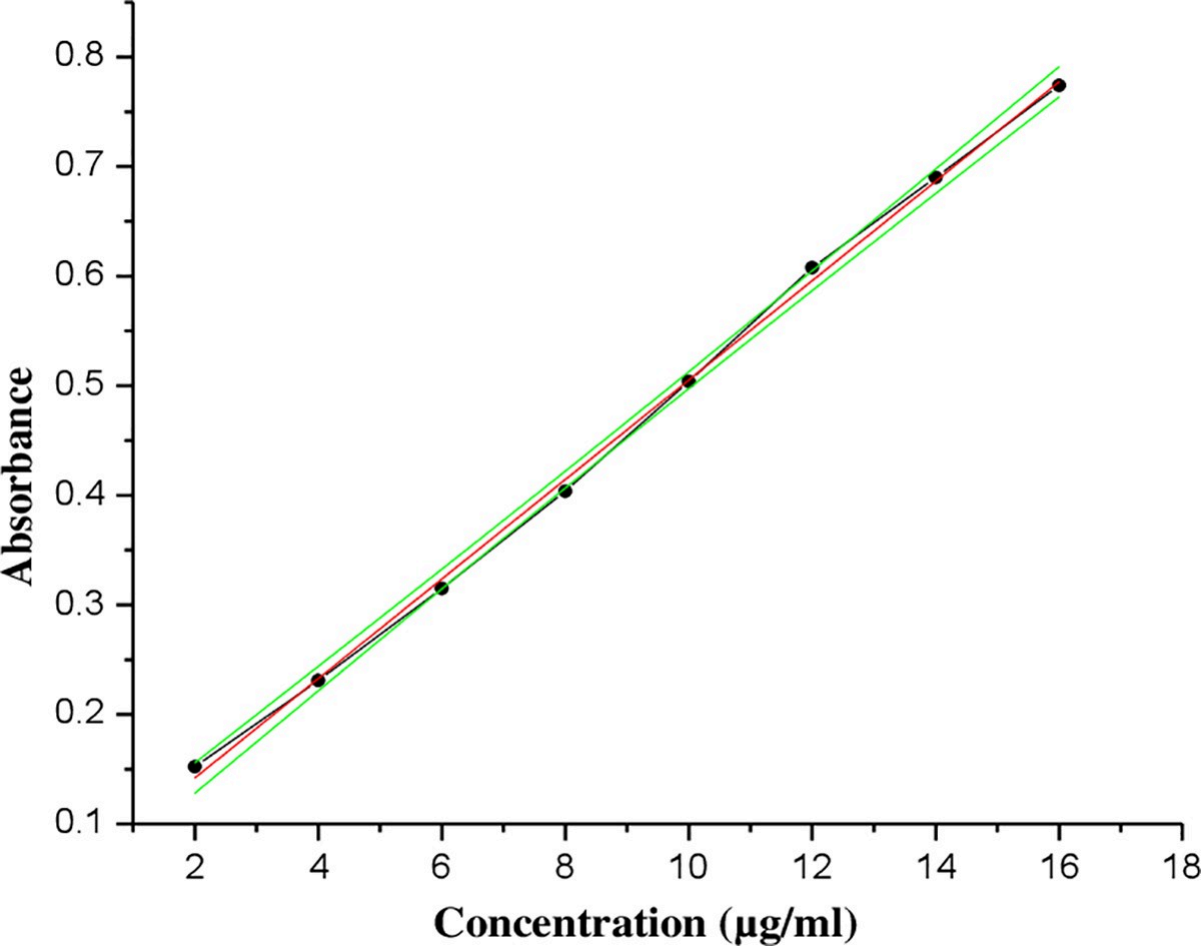
The UV absorption calibration curve of diclofenac sodium in PBS (pH 7.4) at 276 nm with 95% confidence bands for mean, (r^2^ = 0.9987).

Eight different concentrations (2, 4, 6, 8, 10, 12, 14, and 16 μg/ml) were prepared from stock solution containing 100 μg/ml of diclofenac sodium in PBS (pH 7.4). The UV absorbance readings of these solutions were measured at 276 nm using UV/Visible spectrophotometer (PG Instruments Limited, T92+, Leicestershire, UK). PBS (pH 7.4) was used as a blank. The absorbance versus concentration of solutions was plotted and a calibration curve with a linear regression equation of: Y = 0.0454X + 0.0512 (where, Y is the absorbance and X is the concentration in μg/ml) and correlation coefficient of 0.9987 was obtained (Fig 1).

### Physicochemical evaluation of diclofenac sodium gel formulations

The prepared diclofenac sodium gel formulations were evaluated for clarity/transparency, color, scent, texture, consistency, homogeneity, drug content, rheology, clarity, spreadability, extrudability, pH, and kinetics and mechanism of drug release [41, 43–45]. The detailed procedure is put in the S3 Appendix.

### *In vitro* release study of diclofenac sodium from gel preparations

One-gram gel formulation each containing 30 mg of diclofenac sodium was placed on cellulose acetate membrane (pore size 0.45 μm, Sartorius, Goettingen, Germany) and fixed to one end and made water-tight with aid of a rubber band in an apparatus consisting of cylindrical tube with both ends open, with 12.1 mm inner diameter (release area = 115 mm^2^) as a diffusion cell. The tubes were submerged in 1000-ml vessels containing 400 ml PBS (pH 7.4) as receptor medium. The whole assembly was fixed in such a way that the lower end of the cell containing the gel just touched (1–2 mm deep) the diffusion medium. The release test was carried out at a controlled stirring rate of 100 rpm to ensure sink condition and a temperature of 37 ± 1°C by means of water jacket surrounding each cell, based on the facts that the receptor phase is in contact with the deepest skin layers and that the deep body temperature of humans is maintained between 36.2°C and 37.2°C in order to maintain the skin surface at 32°C. An aliquot of 5 mL was withdrawn at specific time intervals up to 720 min, and estimated spectrophotometrically at 276 nm. After each withdrawal, the diffusion medium was replaced with an equal volume of fresh diffusion medium. The cumulative percent release was calculated for each time (in min) interval [41, 43, 45].

### Statistical analysis

All data were analyzed using OriginPro 8.5.1 (OriginLab Corporation, MA, USA) and Excel 2016. The experiments were done in triplicates and the data were presented as the mean ± standard deviation (SD). All data reported in this study were the averages of triplicate determinations. *P* value of less than 0.05 was considered to be evidence for a significant difference, and a Tukey’s test for one-way analysis of variance (ANOVA) was applied when necessary.

## Results and discussion

### Cellulose extraction conditions

In this study, cellulose fibers were extracted with two conditions using 40% formic acid/40% acetic acid, and 80% formic acid/80% acetic acid at the pretreatment stage. It was reported that acetic acid and formic acid can effectively remove lignin and hemicelluloses by cleaving ether bonds between lignin and hemicellulose from different lignocellulosic materials at atmospheric pressure [46, 47].

Hydrogen peroxide in formic acid/acetic acid solution enhanced the delignification process due to the combined effect of the organic acids as solvent and peroxyacid as an oxidizing agent to dissolve the lignin by the action of hydroxonium ion OH^+^ [9, 48]. Bleaching the mass with a solution of hydrogen peroxide in an alkali medium, a chlorine-free bleaching agent, is carried out for the elimination of chromophore compounds to raise the brightness of cellulose, reduce chlorinated organic matter and the effluent odor, which is an important characteristic to produce cellulose derivatives.

Furthermore, a chlorine-free bleaching technique was followed, where oxidation of lignin through cleavage of side chains occurs due to the formation of the perhydroxyl anion (OOH-), a nucleophile intermediate. The action of radicals formed during the bleaching process is responsible to a large extent for the delignification. The bleaching with peroxide causes degradation of the molecule into smaller and water soluble parts [48]. Basically, the chromophore compounds constitute fragments of lignin. Therefore, it is expected that in this process mainly lignin is removed most, preserving the polysaccharides [49].

### Identification and composition of the plant materials

**Fig 2:**
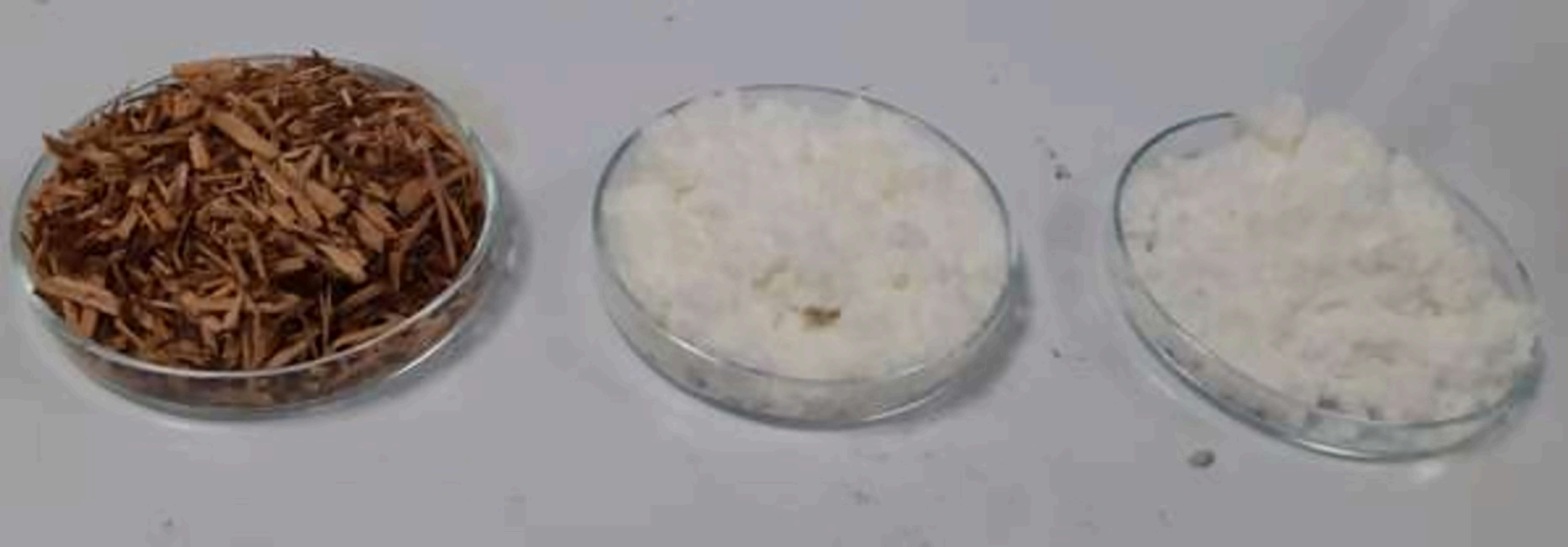
Photographs of untreated khat waste (KW-0), and as-extracted cellulose fibers (C_40_ and C_80_) from left to right.

The cellulose fibers extracted from KW were white, and fluffy and fibrous in nature, and soluble in cuprammonium hydroxide ‘Cuam’ solution, but insoluble in water, acetone, anhydrous ethanol and toluene. A violet-blue color was formed when the extracted cellulose fibers were dispersed in iodinated zinc chloride solution, fulfilling the parameters specified in pharmacopeia [50]. The macroscopic images of the untreated KW, and as-extracted cellulose fibers (C_40_ and C_80_) are shown in Fig 2.

The composition of untreated KW and as-obtained cellulose fibers such as cellulose content, hemicellulose, and lignin is presented in Table 2. Cellulose content increased in the extracted cellulose fibers due to removal of non-cellulosic components [9, 49]. Other studies also reported the increment of cellulose content after chemical treatments of the raw lignocellulosic materials such as coffee silverskin [51] and sugarcane bagasse [52] (Table 2). The brightness, digestibility, and weight of KW cellulose fibers were considerably improved when 80% formic acid and 80% acetic acid (70:30) were used instead of 5% or 10% wt sodium hydroxide at the pretreatment stage. It has been reported that alkaline treatment is very effective in increasing the digestibility of hardwood and agricultural residues with low lignin content [53]. The cellulose content in the untreated khat waste (39%) was comparable with sugarcane bagasse (40–42%) [34, 52], and garlic straw residues (41%) [19], but higher than banana pseudo-stem (24%) [54] and coffee silverskin (31%) [51], and lower than corn husk (45%) [35] (Table 2).

**Table 2:** Chemical composition of untreated KW, and as-obtained cellulose fibers, and other sources.

Plant materials (References)		Composition (%w/w) on dry basis							
		Cellulose	Hemicellulose	Klason lignin	Pectic matters	Fatty and waxy matters	Aqueous extractives	Ash	Others
KW*	0	39.4 ± 0.38	12.75 ± 0.52	28.67 ± 2.46	5.24 ± 0.66	7.87 ± 0.18	3.47 ± 0.35	3.40 ± 0.12	---
	C_40_	82.7 ± 1.43	5.83 ± 0.52	10.89 ± 0.21	0.98 ± 0.07	0.73 ± 0.06	0.66 ± 0.13	1.34 ± 0.18	---
	C_80_	88.4 ± 1.43	3.44 ± 0.48	6.93 ± 0.18	0.88 ± 0.06	0.67 ± 0.11	0.64 ± 0.04	0.97 ± 0.09	---
**Banana pseudo-stem** [54]		23.82	25.69	8.56	5.03	4.25	32.64	7.06	---
**Corn husk** [35]		45.13 (α)	31.15 ± 0.55	14.32 ± 0.23	3.65 ± 0.17	2.20 ± 0.11	2.50 ± 0.07	---	1.05
**Coffee silverskin** [51]	0	31 ± 2%	---	---	---	---	---	2.7 ± 0.8	---
	C	73.60–85.50	---	---	---	---	---		---
**Sugarcane bagasse** [52]	0	42.10	26.10	24.40	---	---	---	1.3	6.1
	C	93.20	3.40	2.30	---	---	---	0.45	0.65
**Sugarcane bagasse** [34]		40.21	25.00	23.90	---	---	---	1.72	---
**Garlic straw residues** [19]	0	41	18	6.3		3.2		10	
	C	86	---	---	---	---	---	9.4	---

*Current study, 0-Raw materials; C-Extracted cellulose; Data were presented as the mean ± SD (n = 3).

Reduction of non-cellulosic materials is observed in the as-extracted cellulose, and increase in the cellulose content, which is in agreement with studies reported elsewhere [19, 49, 55]. The CNCs suspensions were white and turbid in appearance. The yields of CNCs_40_ and CNCs_80_ obtained from C_40_ and C_80_ were 55% and 49%, respectively.

As the lignin content (29%) reported in this study (Table 2) is much higher than that reported in most studies: 14% (sisal fibers) [56], 9% (municipal grass waste) [21], and 9% (lemon seeds) [57], the use of acetic acid and formic acid in the pretreatment step was needed to increase the purity of cellulose and facilitate the removal of lignin as well as digestibility, and thereby enhance the whiteness of the pulps. However, in a preliminary work during extraction of cellulose from other lignocellulosic materials with low lignin content, the whiteness, cellulose content as well as the degree of crystallinity increased significantly without such additional step. Different studies which employ sodium hydroxide in the pretreatment stage use chlorine containing bleaching agents mainly sodium chlorite solution [21, 57–60] during isolation of CNCs, however, a chlorine-free bleaching solvent (alkaline hydrogen peroxide) was employed in our study.

### Chemical functionality studies

**Fig 3:**
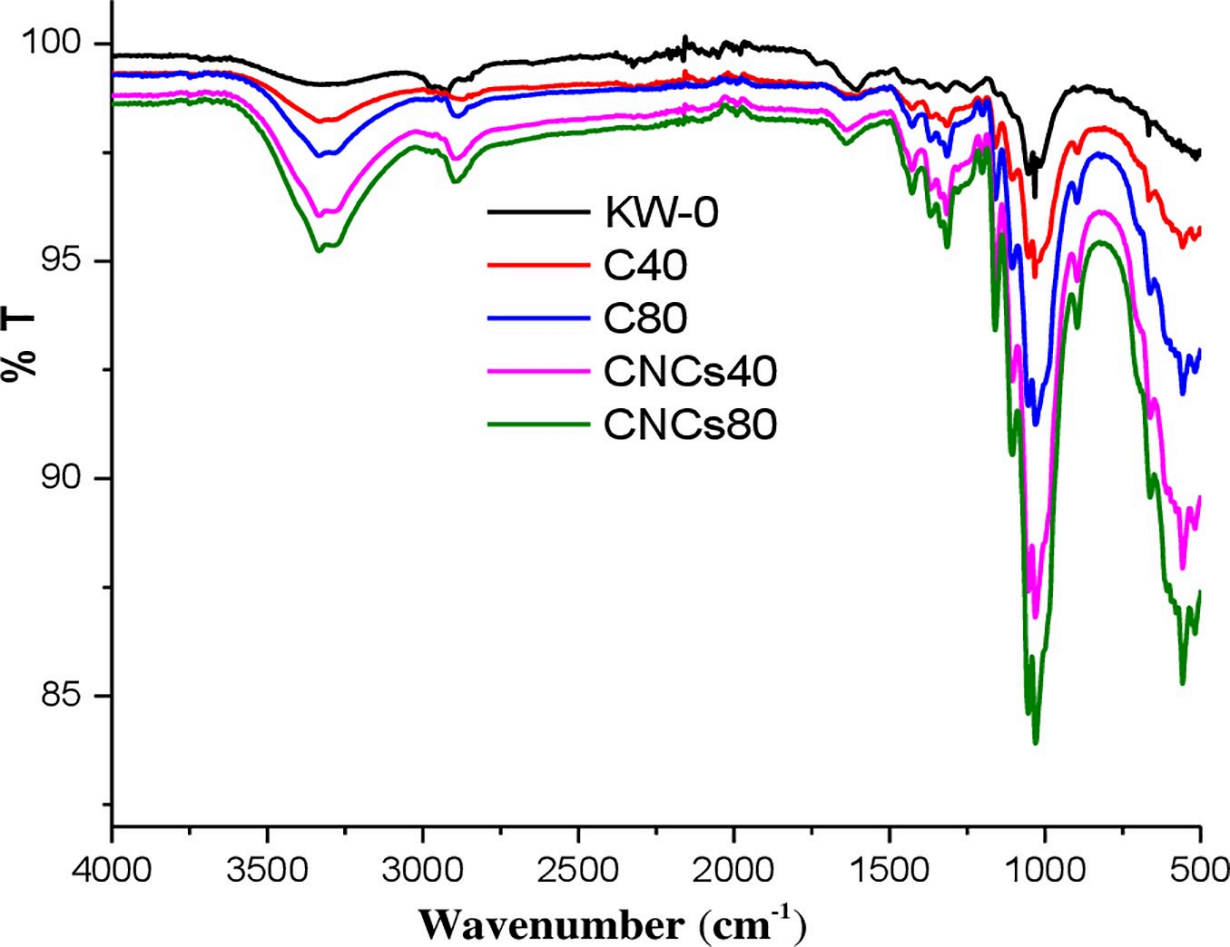
FTIR spectra of untreated KW, as-obtained cellulose fibers (C_40_ and C_80_) and CNCs (CNCs_40_ and CNCs_80_). (Key: KW-0: untreated khat waste; C_40_ and C_80_: cellulose fibers obtained from khat waste with 40% formic acid and 40% acetic acid, and 80% formic acid and 80% acetic acid, respectively at the pretreatment stage; CNCs_40_ and CNCs_80_: cellulose nanocrystals isolated from C_40_ and C_80_, respectively).

Fig 3 illustrates the FTIR spectra of untreated KW, as-obtained cellulose fibers, and CNCs. FTIR spectroscopy revealed the similarities of all spectra of cellulose fibers and CNCs, showing similar chemical composition among the samples [61]. The broad absorption band around 3340 cm^*‐*1^ is due to the stretching vibrations of the OH groups, indicating the hydrophilic tendency of the materials. The weak band around 2900 cm^*‐*1^ is due to the asymmetric stretching vibration of the CH_2_ bond [62]. Furthermore, the appearance of a peak at ~1645 cm^*‐*1^ in all the spectra shows absorption of water by the materials [40].

The untreated KW indicated typical peaks around 1736 cm^-1^, 1516 cm^-1^, and 1235 cm^-1^. The disappearance of the peak around 1736 cm^-1^ showed the cleavage of the linkages between ferulic acid or p-coumaric acid or (p-) hydroxycinnamic acids and lignin during chemical treatment. The absence of the bands around 1516 cm^-1^ and 1235 cm^-1^ in the cellulose fibers and CNCs indicated that lignin functional groups such as aromatic rings were dissociated and dissolved. Similar findings were also reported by different research groups indicating removal of non-cellulosic materials using different treatment conditions [9, 51, 63, 64].

The absorption band at around 1428 cm^-1^ is associated with intermolecular hydrogenattraction at the C_6_ group. The peak around 1323 cm^-1^ region of the spectra is attributed to the bending vibration of the CH and CO groups of aromatic ring in the materials [61]. The peak around 896 cm^*‐*1^ is due to C_1_H rocking vibration of cellulose (β-glycosidic linkages). The bands around 3340, 2900, 1428, 1323, and 896 cm^*‐*1^ in all cellulose and CNCs spectra are associated with the characteristics of cellulose I, showing that the acid hydrolysis did not affect the chemical structure of the cellulosic fragments [65, 66].

### Crystallinity analysis

**Fig 4:**
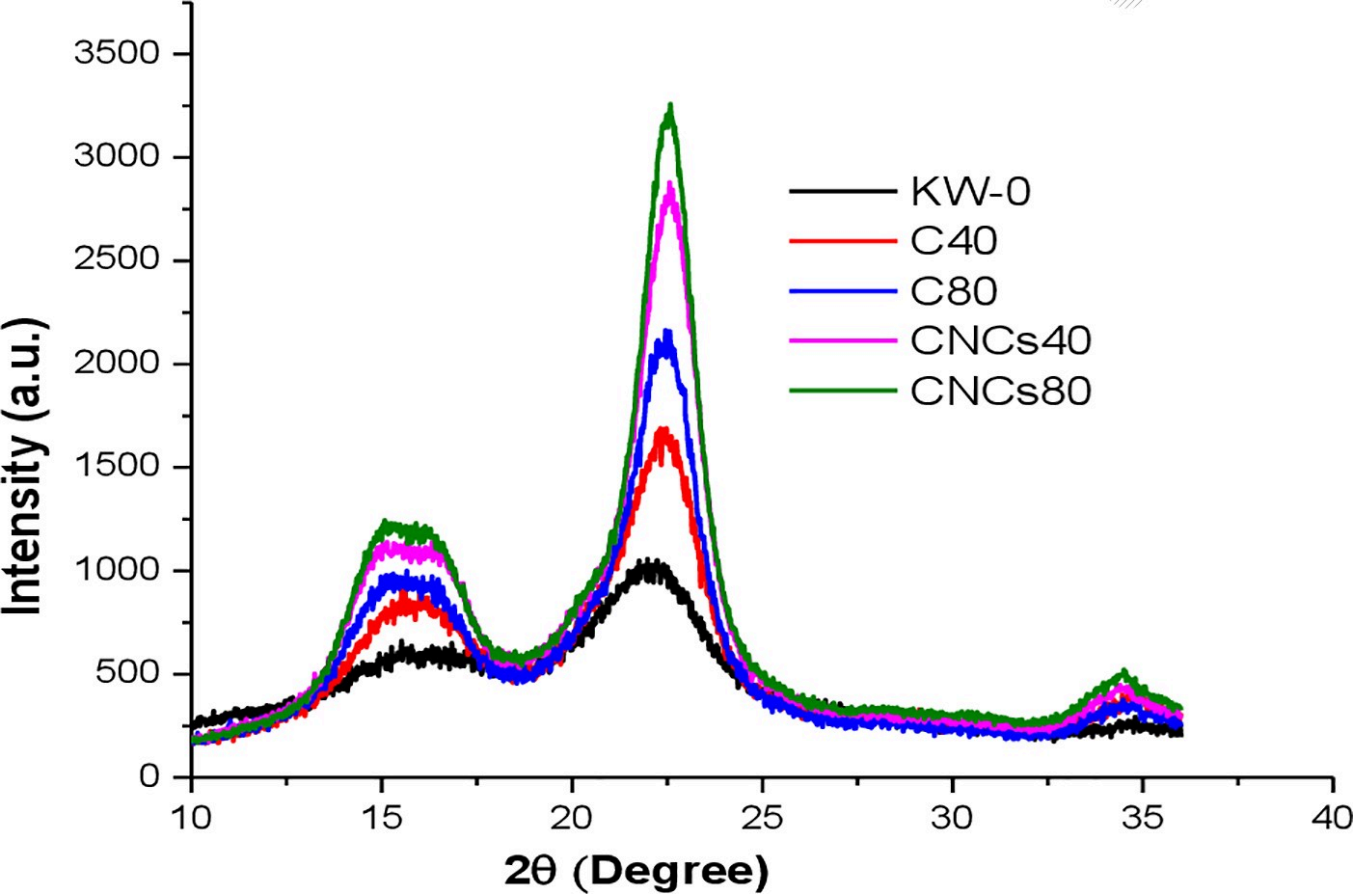
XRD of untreated KW, as-obtained cellulose fibers (C_40_ and C_80_) and CNCs (CNCs_40_ and CNCs_80_). (Key: KW-0: untreated khat waste; C_40_ and C_80_: cellulose fibers obtained from khat waste with 40% formic acid and 40% acetic acid, and 80% formic acid and 80% acetic acid, respectively at the pretreatment stage; CNCs_40_ and CNCs_80_: cellulose nanocrystals isolated from C_40_ and C_80_, respectively).

The as-isolated CNCs_40_ and CNCs_80_ like their cellulose precursors and untreated KW (KW-0) displayed a typical crystal lattice of Cellulose I, with the main diffraction signals around 2θ values of 15°, 16°, 22° and 34° with assigned crystallographic plane of 1‐10, 110, 200 and 040, respectively after deconvolution using Gaussian profile as reported elsewhere [61, 64, 67]. The XRD patterns of the untreated KW, as-obtained cellulose and CNCs are shown in Fig 4.

CNCs_80_ (82.84%) exhibited almost comparable CrI with CNCs_40_ (81.60%), but much higher than that of untreated KW (56.89%). Removal of non-cellulosic was also confirmed by the increment of CrI of cellulose fibers when compared to the untreated KW (Fig 4 and S1 Table). Generally, the CrIs increased in both isolated CNCs when compared to their cellulose precursors. Such an increment of crystallinity was due to the removal of hemicellulose and lignin existing in amorphous regions, and during the hydrolysis process [68]. The CrIs of CNCs reported in this study were higher when compared to CrIs of CNCs isolated from others sources such as passion fruit peels waste (77.96%) [14], pineapple crown waste (73%) [69], seaweed (60%) [68], and pueraria root residue (60%) [70].

The τ values of the CNCs_40_ and CNCs_80_ were 5.466 nm and 5.633 nm, and X values were 0.630 and 0.672, respectively. S1 Table shows different parameters obtained from the (deconvoluted) XRD of untreated KW, as-extracted cellulose, and CNCs_40_ and CNCs_80_. The d-spacings of the all the samples ranged from 0.589‐0.608, 0.523‐0.565, 0.385‐0.401, and 0.258‐0.262 for the planes of 1‐10, 110, 200, and 040, respectively as shown in S1 Table. All the samples including CNCs_40_ and CNCs_80_ are all I_β_-type cellulose, as confirmed by XRD patterns, d-spacing values, and the negative numbers of the Z-Values [71, 72] (S1 Table).

### Morphological, dimensional and zeta potential investigation

**Fig 5:**
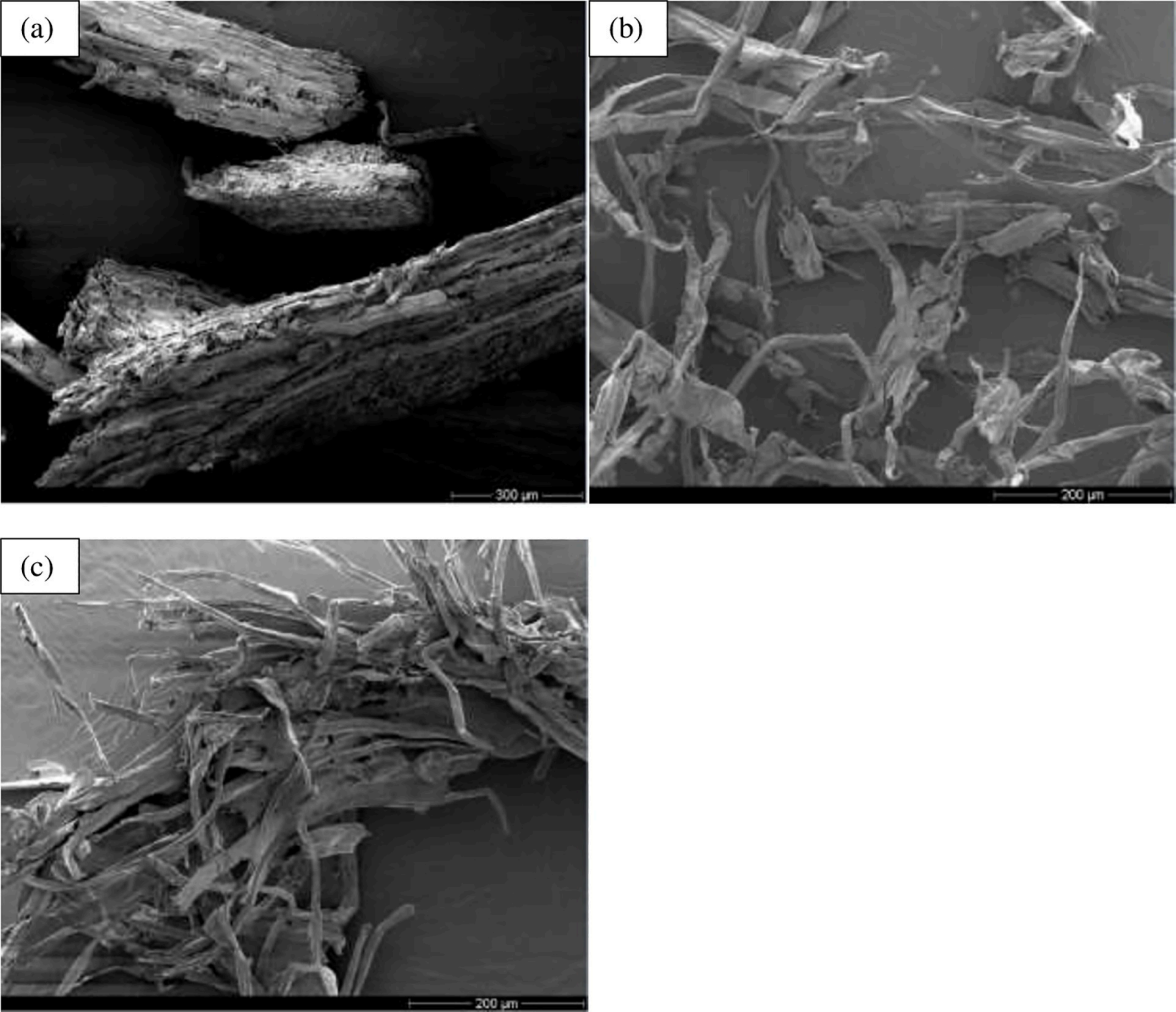
Scanning electron micrographs of (a) untreated khat waste (KW-0, with scale bar of 300 μm), and as-obtained cellulose fibers: (b) C_40,_ and (c) C_80_ (with scale bar of 200 μm).

Pretreatment using formic acid and acetic acid, followed with delignification/bleaching stages resulted in changes of chemical composition of the as-obtained cellulose fibers and the structure of the fibers surfaces. The surface morphology of untreated KW and as-extracted cellulose is investigated with SEM as shown in Fig 5. SEM images indicated that untreated KW existed as rough and compact fibrillar packing displaying lot of non-cellulosic components such as lignin scattered over the cellulosic fiber surface, acting as cementing materials, consistent with other finding [73]. The cellulose bundles are composed of individual fibers linked together by the massive cementing material. Most of the lignin and hemicellulose were hydrolyzed during chemical treatments. The average diameters of the as-extracted cellulose fibers (16 and 28 μm) are much smaller than that of untreated KW (263 μm) (Table 3), indicating the removal of non-cellulosic components during the treatment conditions. In a study reported elsewhere, the diameter of untreated sisal fibers ranged from 100–500 μm and the diameter of the extracted cellulose fiber was reduced to 7–31 μm [74].

**Table 3:** Average diameters (SEM) of the untreated KW, and as-extracted cellulose fibers (C_40_ and C_80_), and TEM dimensional analysis, DLS and Zeta potential values of the CNCs.

CNCs	Length (L) range; L_average_ (nm)	Diameter (D) range; D_average_	Aspect ratio	Hydrodynamic size (nm); PDI (from DLS)	ZP (mV)
**KW-0**	--	263.04 ± 45.37 μm	--	--	**--**
**C**_**40**_	--	27.97 ± 12.20 μm	--	--	**--**
**C**_**80**_	--	16.08 ± 3.02 μm	--	--	**--**
**CNCs**_**40**_	162.96 ± 26.04	7.17 ± 1.86 nm	22.73	362.8; 0.461	‐75.3
**CNCs**_**80**_	101.55 ± 20.53	7.91 ± 2.56 nm	12.84	222.8; 0.297	‐45.7

(Key:- KW-0: untreated khat waste; C_40_ and C_80_: cellulose fibers obtained from khat waste with 40% formic acid and 40% acetic acid, and 80% formic acid and 80% acetic acid, respectively at the pretreatment stage; CNCs_40_ and CNCs_80_: cellulose nanocrystals isolated from C_40_ and C_80_, respectively; D_average_-average diameter of untreated KW, cellulose fibers, and CNCs estimated using ImageJ Software; L_average_-average length of CNCs; PDI: Polydispersity index; ZP: Zeta potential). Data were presented as the mean ± SD (n > 15).

**Fig 6:**
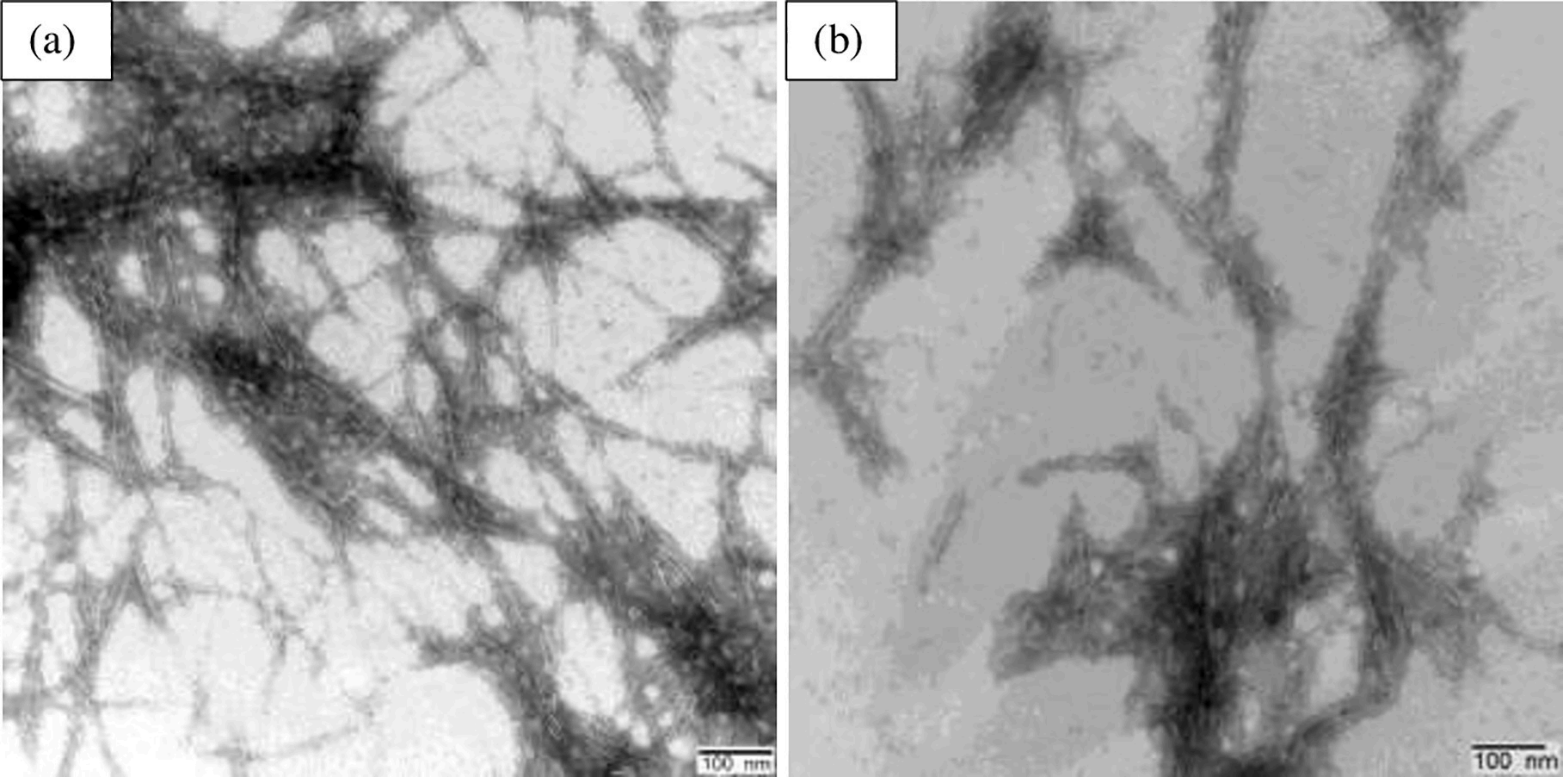
Transmission electron micrographs of CNCs isolated from KW: CNCs_40_ (a) and CNCs_80_ (b) (Bar scale: 100 nm).

TEM images show appearance of needle-shaped CNCs on the acid hydrolysis and sonication with a scale bar of 200 nm (Fig 6). From the TEM analysis, the length and diameter of the CNCs isolated from the byproducts ranged from 106.78‐193.06 nm and 5.16‐11.79 nm, respectively. Furthermore, the average aspect ratio of the CNCs ranged from 17.32‐36.68 (Table 3).

The DLS results also supported the TEM results that the isolated CNCs were in nanoscale range, and their hydrodynamic size were 362.8 nm and 222.8 nm for CNCs_40_ and CNCs_80_, respectively (Table 3). The DLS and TEM results suggested that increasing the concentration of weak acids at the pretreatment stage contributed for reduced particle size and aspect ratios of the isolated CNCs. The ZP of the CNCs suspensions ranged from ‐45.7 and ‐75.3 mV in neutral water and 0.1 N PBS (S1 Fig and Table 3), and resulted in stable colloidal suspensions as the absolute values obtained are higher than ‐15 mV which is the minimum value to represent the onset of agglomeration [33, 75, 76]. ZP of CNCs at the values near or lower than ‐20 mV at low concentrations remain stable [77].

### Thermal properties

**Fig 7:**
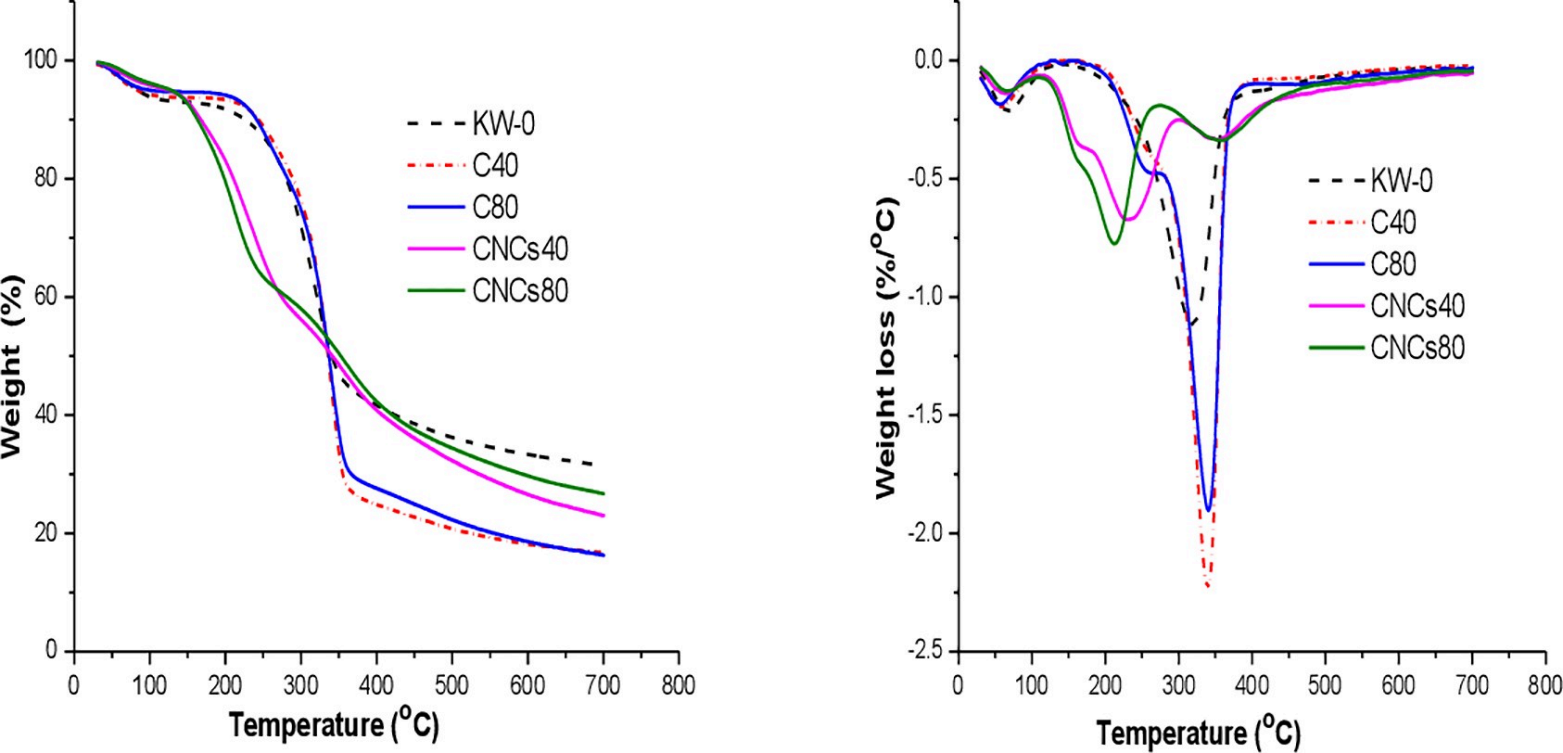
Thermal degradation behaviors: TGA (a) and DTG (b) of untreated KW (KW-0), as-obtained cellulose fibers (C_40_ and C_80_) and CNCs (CNCs_40_ and CNCs_80_). (Key:- KW-0: untreated khat waste; C_40_ and C_80_: cellulose fibers obtained from khat waste with 40% formic acid and 40% acetic acid, and 80% formic acid and 80% acetic acid, respectively at the pretreatment stage; CNCs_40_ and CNCs_80_: cellulose nanocrystals isolated from C_40_ and C_80_, respectively).

Fig 7 demonstrates the thermogravimetric (TG) and derivative thermogravimetric (DTG) curves of the untreated KW, as-extracted cellulose fibers and CNCs. The thermal degradation data (ΔT), (T_10%_), (T_50%_), weight loss (rate) at each region (%), the residual weight at 500 and 700°C as well as the peak degradation temperatures (T_max_) are listed in S2 Table. The small weight loss (3.85‐6.38%) in the region 30‐120°C is mainly due to loss of water adsorbed to the materials [12, 15, 60, 70, 78]. The as-obtained cellulose fibers and CNCs contained relatively lower moisture than respective untreated KW as shown in S2 Table, which might be due to the removal of hydrophilic components such as hemicellulose and lignin in cellulose fibers and CNCs [73].

The DTG thermograms of untreated KW and as-extracted cellulose fibers showed a sudden reduction in weight loss around 189 ^o^C, mainly due to the loss of hemicellulose. The acetyl groups of hemicellulose contributed for the low thermal stability. A major weight loss was observed at T_max_ 320–400 ^o^C due to depolymerisation, dehydration and decomposition of the glycosidic units of cellulose [33]. There was poor/no identifiable peak of lignin in the TGA/DTG thermograms of the samples including untreated KW due to its slow and resistant decomposition ranging from ambient temperature to 700 ^o^C [65].

The CNCs exhibited a weight loss of 34‐38% in the region at T_max_ 213 and 232°C due to degradation of surface sulfate groups lowering the activation energy and large specific surface area, when compared to the cellulose precursors (weight loss of 67‐69% at T_max_ of 340°C. Another decomposition step with a weight loss of 22% exhibited at T_max_ ranging from ~360°C (the major cellulose degradation temperature), due to breakdown of the interior non-sulfated cellulose crystals [60]. The CNCs also exhibited lower maximum weight loss rates (0.6726‐0.7744%/°C) in the sulfated cellulose groups than the cellulose counterparts (1.9019–2.2346%/°C). The char residues at 550°C and 700°C of the isolated CNCs showed higher values than cellulose counterparts because of a dehydration effect of the sulfate group as flame retardants [20, 60, 78, 79].

### Evaluation of diclofenac sodium gel formulations

Five different topical diclofenac sodium gel formulations containing CNCs were prepared as a reinforcing material in CMC gel base, however, the sixth one did not contain CNCs_40_. CNCs_40_ was selected as it had better aspect ratio and colloidal stability when compared to CNCs_80._ All diclofenac sodium gel formulations were smoothly spreadable without any solid or gritty particles, homogeneous, and transparent in physical appearance. The presence of triethanolamine in the medicated gel formulations improved the clarity when compared with the polymer gel bases, suggesting the solubility of the drug in the gel matrix. Triethanolamine was incorporated to adjust the pH and to increase the solubility of the drug in the gel formulations [45, 80].

The pH values of diclofenac sodium gel formulations varied from 6.81 ± 0.042–7.43 ± 0.033, which is physiologically acceptable pH range and free from any skin irritation. The content uniformity of diclofenac sodium in all gel formulations ranged from 98.76 ± 0.41–101.2 ± 0.52% which are within the acceptable limits [50]. In the spreadability test, the diameters of diclofenac sodium gel formulations ranged from 54 ± 3.4–72 ± 2.5 mm. Viscosity is an important physical parameter for characterizing the gel formulations as it affects the extrudability, spreadability, and release of drug and other physicochemical properties of gel preparations. The viscosity of the gel formulations declined proving shear-thinning flow when the shear rate varied from 0.5 to 200 rpm (67 to 6667 sec^-1^) (S2 Fig).

### *In vitro* release and kinetics of diclofenac sodium

**Fig 8:**
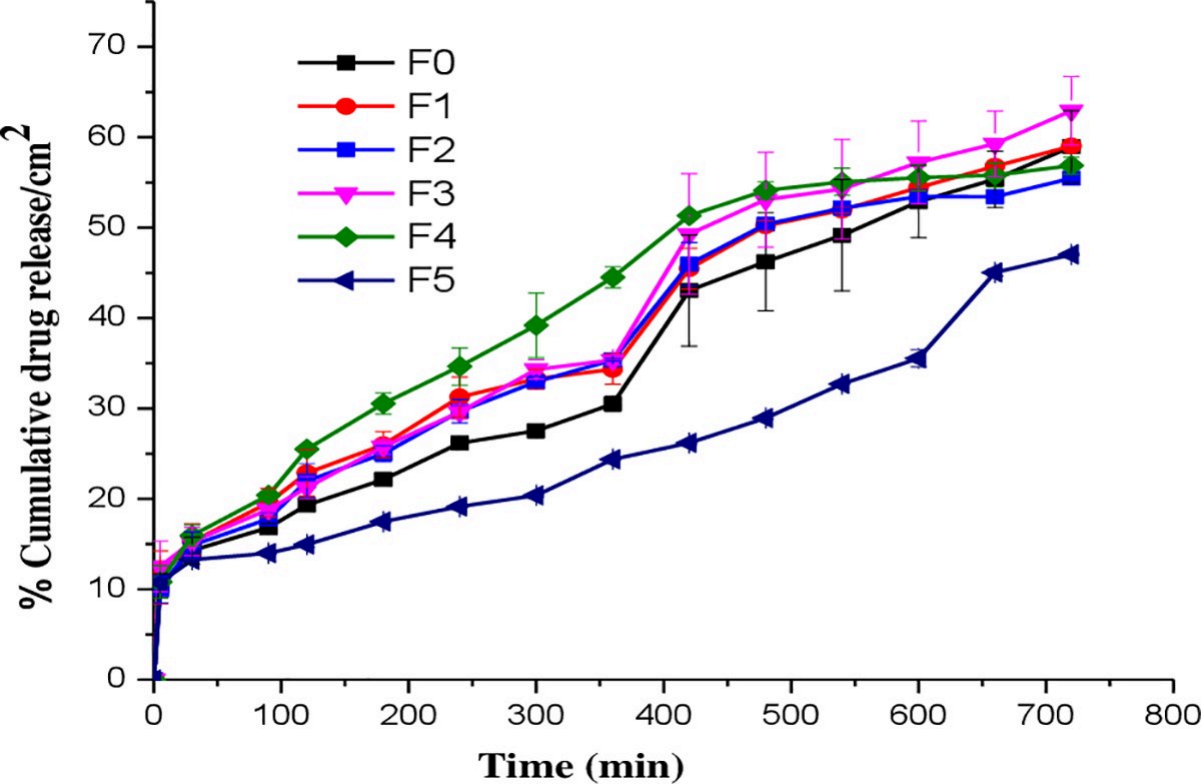
*In vitro* diclofenac sodium release profiles from gel formulations (F0-F5).

From the release profiles of diclofenac sodium from the six gel formulations as depicted in Fig 8, it is observed that, initially (~1 h), the drug was released rapidly (burst effect) followed by a slow release for the rest of the 12 h study period. The initial burst effect could be due to the release of the drug to the surface of the immediate barrier membrane. The results show that when the concentration of CNCs in the gel formulations increased from 0.25 to 2% (w/w), the percent of diclofenac sodium released into the buffered medium gradually decreased to 47%/cm^2^.

The drug release pattern best followed Higuchi kinetic models when the data were analyzed and compared with zero order, and first order kinetics, as confirmed by the good correlation coefficients (S3 Table). This finding indicates that the rate-controlling stage in the release process is the diffusion of the dissolved drug through the vehicle network to the external medium. As the viscosity of gels increase, the release the drug becomes slower by extending dissolution time and prolonging drug diffusion through the gel matrix. The viscosity of vehicles may play an important role in controlling the drug release when the drug diffusion through the vehicle is a rate limiting step.

## Conclusions

The present research includes successful extraction of cellulose fibers and CNCs from abundantly available KW using two chlorine-free isolation conditions. Removal of non-cellulosic materials such as hemicellulose and lignin were confirmed by the FTIR, XRD and SEM studies. The untreated KW, as-obtained cellulose fibers, and CNCs exhibited the typical peaks of Cellulose I_β_ around 15, 16, 22 and 34° 2θ, as confirmed by XRD pattern, d-spacings, and Z-values. The increment of formic acid/acetic acid concentration from 40% to 80% did not significantly increase CrI of the CNCs after acid hydrolysis. Additionally, higher/comparable yield, aspect ratio, colloidal and thermal stability were observed in CNCs_40_. The CNCs can be used as a reinforcing material to increase the gel strength, and enhance sustained delivery of drugs. The findings suggest that cellulose fibers and CNCs can be obtained from KW as alternative source using ecofriendly method.

## Supporting information

S1 Appendix Methods for determination of the composition of the plant materials. (DOCX)

S2 Appendix Physicochemical evaluation of the medicated gel formulations. (DOCX)

S3 Appendix Equations for the determination of different parameters from (deconvoluted) XRD diffractograms. (DOCX)

S1 Fig Zeta potential of a) CNCs40 and b) CNCs80.
(DOCX)

S2 Fig Rheological profiles of diclofenac sodium gel formulations (F0-F5). (DOCX)

S1 Table Properties obtained from the (deconvoluted) XRD of untreated KW, as-obtained cellulose fibers, and CNCs. (DOCX)

S2 Table Summary of thermal properties of untreated khat waste, obtained cellulose, and CNCs. (DOCX)

S3 Table *In vitro* diclofenac sodium release characteristics from the gel formulations and fitting models. (DOCX)
