# Supplementary material for: Valorization of khat (Catha edulis) waste for the production of cellulose fibers and nanocrystals
Source: PLoS One. 2021 Feb 9;16(2):e0246794. doi: 10.1371/journal.pone.0246794 (PMC7872298; doi:10.1371/journal.pone.0246794)
Supplement: S1 Appendix — (DOCX) [file pone.0246794.s001.docx]

## S1 Appendix. Methods for determination of the composition of the plant materials

The constituents of the untreated KW as well as as-obtained cellulose fibers such as lignin, hemicellulose and others were determined according to the methods stated elsewhere [1–5].

### Klason lignin

To determine klason lignin, methods reported elsewhere were followed [2,3,5]. First, defatted and dewaxed samples were treated with 72% wt/wt H_2_SO_4_ in a ratio of 1:20 by adding drop-wise with constant stirring. Then, the mixture was allowed to stand for 2 h and then diluted to 3% acid concentration and boiled for 4 h under reflux, and allowed to stand overnight. The lignin was filtered on a pre-weighed filter paper and washed with hot distilled water till neutrality. The lignin was then dried at 105 °C for 6 h and gravimetrically estimated after subtracting the ash value.

### Hemicellulose content

The amount of hemicellulose content was determined according to the method described elsewhere [4]. The defatted and dewaxed samples (1:10) were treated after drying using 0.5 M of NaOH solution at 80 °C for 3.5 h. Then, the samples were repeatedly washed with distilled water until the pH was neutral and dried to a constant weight. The difference between the sample weight before and after this treatment was taken as the amount of hemicellulose.

### Water soluble components

To estimate water soluble components, the plant materials were treated with boiling distilled water for 2 h on hot plate, filtered and dried at 105 °C until a constant weight was obtained. % Water soluble components = W_1_ x 100%/W_0_, where W_1_ is the loss in weight and W_0_ is the initial weight of the sample (before treatment) [3].

### Fatty and waxy matters

To estimate fatty and waxy matters, the sample was immersed in an n-hexane–alcohol mixture (2:1, v/v), in the solid to liquor ratio of 1:100, and then allowed to stand for 10 h with occasional stirring. The sample was then washed with fresh n-hexane–alcohol mixture and finally with alcohol. Then, the sample was dried at 105 °C until a constant weight was obtained. The amount of fatty and waxy matter was estimated as follows: % Fatty and waxy matters = W_1_ x 100%/W_0_, where W_1_ is the loss in weight and W_0_ is the initial weight of the sample (before treatment) [3].

### The pectic matter

To determine the pectic matter, the defatted and dewaxed sample was heated in 0.5% ammonium oxalate solution, in the solid to liquor ratio of 1:100, at 70–80 °C for 3 days in a heating mantle. The sample was filtered, washed thoroughly with hot distilled water and then dried at 105 °C until a constant weight was obtained. The percentage of pectic matters was then calculated as: % Pectic matters = W_1_ x 100%/W_0_, where W_1_ is the loss in weight and W_0_ is the initial weight of the sample (before treatment) [3].

### The cellulose content

The content of cellulose in the extracted cellulose was calculated by difference, assuming that water soluble extractives, hemicellulose, lignin, fatty and waxy matter, and pectic matter, and cellulose are the only components [2].

# References

1. Gabriel T, Belete A, Syrowatka F, Neubert RHH, Gebre-Mariam T. Extraction and characterization of celluloses from various plant byproducts. Int J Biol Macromol. 2020;158: 1248–1258. doi:10.1016/j.ijbiomac.2020.04.264

2. Abdel-Halim ES. Chemical modification of cellulose extracted from sugarcane bagasse: Preparation of hydroxyethyl cellulose. Arab J Chem. 2014;7: 362–371. doi:10.1016/j.arabjc.2013.05.006

3. Yeasmin MS, Mondal MIH. Synthesis of highly substituted carboxymethyl cellulose depending on cellulose particle size. Int J Biol Macromol. 2015;80: 725–731. doi:10.1016/j.ijbiomac.2015.07.040

4. Lin L, Yan R, Liu Y, Jiang W. In-depth investigation of enzymatic hydrolysis of biomass wastes based on three major components: Cellulose, hemicellulose and lignin. Bioresour Technol. 2010;101: 8217–8223. doi:10.1016/j.biortech.2010.05.084

5. Smyth M, García A, Rader C, Foster EJ, Bras J. Extraction and process analysis of high aspect ratio cellulose nanocrystals from corn (Zea mays) agricultural residue. Ind Crops Prod. 2017;108: 257–266. doi:10.1016/j.indcrop.2017.06.006
