# Supplementary material for: Valorization of khat (Catha edulis) waste for the production of cellulose fibers and nanocrystals
Source: PLoS One. 2021 Feb 9;16(2):e0246794. doi: 10.1371/journal.pone.0246794 (PMC7872298; doi:10.1371/journal.pone.0246794)
Supplement: S2 Appendix — (DOCX) [file pone.0246794.s002.docx]

## S2 Appendix. Physicochemical evaluation of the medicated gel formulations

### Visual appearance and cosmetic qualities

All the prepared gels were physically inspected for clarity/transparency, color, scent, texture, consistency, and homogeneity after the gels have been set in the containers. The prepared gels were also evaluated for the presence of any particles or aggregates [1].

### pH

The pH of each gel formulation was measured using a pH meter (model PH-210, HANNA instruments, Portugal). The average of three readings was recorded as pH value.

### Drug content determination

Specific quantity (100 mg) of prepared gels was withdrawn at random from three different sampling points from each batch and dissolved in 50 ml of PBS (pH 7.4) and 2.5 ml aliquot was diluted in 25 ml volumetric flask. The volumetric flask containing the gel solution was vigorously mixed to ensure complete solubility of the formulation ingredients. This solution was filtered and the concentration was estimated spectrophotometrically at 276 nm using PBS (pH 7.4) as blank [2,3].

### Rheological studies

The viscosity of the medicated gel formulations at different concentrations was determined by cone and plate viscometer (CAP2000+, USA) at different shear rates ranging from 5 to 500 rpm at room temperature.

### Gel clarity

In addition to visual inspection, gel clarity was also determined by placing a sample of polymer gel or gel preparation in a disposable cuvette and measuring the absorbance at 700 nm using a spectrophotometer against water blank [4].

### Spreadability of ibuprofen gel formulations

Concentric circles of different radii were drawn on graph paper and a 400 cm^2^ glass plate was fixed onto it. Gel (1.0 g) was transferred to the center of the lower plate and spread over a diameter of 2.4 cm. Another 400 cm^2^ glass plate of 185 g was placed gently on the gel and a standardized weight of 170 g was allowed to rest on the upper glass plate for 3 min. The increase in the diameter due to gel spreading was recorded [1,3].

## Kinetics and mechanism of drug release

To analyze the mechanism of drug release from the topical gel, the release data were fitted to the following equations:

1. **Zero – order equation:**

$Q= Q_{o}-K_{o}t$

where, $Q_{o}$ is the amount of drug present initially, $Q$ is the amount of drug remaining at time t, and $K_{o}$ is the zero – order release rate.

1. **First – order equation:**

$\ln Q=\ln Q_{o}-K_{1}t$

where, $Q_{o}$ is the amount of drug present initially, $Q$ is the amount of drug remaining at time t, and$K_{1}$ is the first – order release rate constant.

1. **Higuchi’s equation:**

$Q=2C_{o}\sqrt{(D_{app}\times t/\pi)}$

where, $Q$ is the amount of drug release per unit area at time $t$ (mg cm^-2^), $t$ is the time after the application (sec), $C_{o}$ is the initial drug concentration in the donor chamber (mg cm^-3^), $\pi$ is a constant and $D_{app}$ is the apparent diffusion coefficient (cm^2^ sec^-1^); $D_{app}$ is calculated from the slope$K$ (apparent release rate) of the linear plot of $Q$ versus t^1/2^; hence,

$D_{app}=\frac{K^{2}\pi}{4{C_{o}}^{2}}$

# References

[1] M.G.B. Dantas, S.A.G.B. Reis, C.M.D. Damasceno, L.A. Rolim, P.J. Rolim-Neto, F.O. Carvalho, Quintans-Junior, L. José, Almeida, Silva, J.R.G. da S. Almeida, Development and Evaluation of Stability of a Gel Formulation Containing the Monoterpene Borneol, Sci. World J. 2016 (2016) 1–4. https://doi.org/http://dx.doi.org/10.1155/2016/7394685.

[2] R. Aiyalu, A. Govindarjan, A. Ramasamy, Formulation and evaluation of topical herbal gel for the treatment of arthritis in animal model, Brazilian J. Pharm. Sci. 52 (2016) 493–507. https://doi.org/10.1590/s1984-82502016000300015.

[3] T. Gabriel, A. Belete, T. Gebre-Mariam, Preparation and evaluation of carboxymethyl enset and cassava starches as pharmaceutical gelling agents, J. Drug Deliv. Ther. 3 (2013) 1–10.

[4] O.S. Kittipongpatana, S. Burapadaja, N. Kittipongpatana, Carboxymethyl mungbean starch as a new pharmaceutical gelling agent for topical preparation, Drug Dev. Ind. Pharm. 35 (2009) 34–42. https://doi.org/10.1080/03639040802144229.
