# Supplementary material for: Valorization of khat (Catha edulis) waste for the production of cellulose fibers and nanocrystals
Source: PLoS One. 2021 Feb 9;16(2):e0246794. doi: 10.1371/journal.pone.0246794 (PMC7872298; doi:10.1371/journal.pone.0246794)
Supplement: S3 Appendix — (DOCX) [file pone.0246794.s003.docx]

## S3 Appendix. Equations for the determination of different parameters from (deconvoluted) XRD diffractograms

X-ray diffraction of the cellulose fibers and as-isolated CNCs were deconvoluted following Gaussian profile, and parameters such as d-spacings (d), apparent crystallite size or thickness for the 200 plane (τ_200_), fractional variation in the plane spacing for the 200 plane (Δd/d)_200_, the proportion of crystallite interior chains for the 200 plane (X_200_), and Z-values were determined using equations described elsewhere [1–4].

The d-spacings were calculated using the Bragg equation:

$d=\frac{\lambda}{2sin\theta}$ -----------------------------------------1

Where, λ is the wavelength of the incident X-rays, d is the interplanar spacing of the crystal and θ is the angle of incidence.

The average thickness of cellulose crystallites was estimated from the XRD patterns by using Scherrer’s equation:

$\tau=\frac{\kappa\lambda}{\beta_{\frac{1}{2}}cos\theta}$-----------------------------------------2

where τ is the crystallite dimension/size, κ is the correction factor and usually taken to be 0.94, λ is the radiation wavelength (0.1542 nm), $\theta$ is the diffraction angle corresponding to 200 plane and β_1/2_ is the peak width at half maximum intensity.

The proportion of crystallite interior chains (X) is calculated using the equation:

$X=\frac{{(\tau-2h)}^{2}}{\tau^{2}}$------------------------------------------3

where $\tau$ is the apparent crystallite size for the reflection of plane (200), and h = 0.57 nm is the layer thickness of the surface chain.

Also, the fractional variation in the plane spacing Δd/d for the 200 plane was calculated following the equation:

$\frac{\Delta d}{d}= \frac{\beta}{2tan\Theta}$-----------------------------------------4

The Z-value indicates whether cellulose is I_α_ or I_β_. The function that discriminates between I_α_ or I_β_ is given by equation:

Z$=1693d1-902d2-549$-------------------5

Where, d_1_ is the d-spacing of the (1-10) peak and d_2_ is the d-spacing of the (110) peak.

# References

1. Popescu M, Popescu C, Lisa G, Sakata Y. Evaluation of morphological and chemical aspects of different wood species by spectroscopy and thermal methods. J Mol Struct. 2011;988: 65–72. doi:10.1016/j.molstruc.2010.12.004

2. Aguayo MG, Pérez AF, Reyes G, Oviedo C, Gacitúa W, Gonzalez R, et al. Isolation and characterization of cellulose nanocrystals from rejected fibers originated in the Kraft Pulping process. Polymers (Basel). 2018;10: 1145–1156. doi:10.3390/polym10101145

3. Poletto M, Ornaghi Júnior HL, Zattera AJ. Native cellulose: Structure, characterization and thermal properties. Materials (Basel). 2014;7: 6105–6119. doi:10.3390/ma7096105

4. Gabriel T, Belete A, Syrowatka F, Neubert RHH, Gebre-Mariam T. Extraction and characterization of celluloses from various plant byproducts. Int J Biol Macromol. 2020;158: 1248–1258. doi:10.1016/j.ijbiomac.2020.04.264
