# Supplementary figures and images for: Valorization of khat (Catha edulis) waste for the production of cellulose fibers and nanocrystals
Source: PLoS One. 2021 Feb 9;16(2):e0246794. doi: 10.1371/journal.pone.0246794 (PMC7872298; doi:10.1371/journal.pone.0246794)

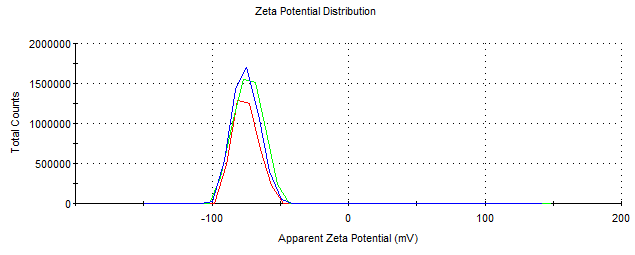


# a)


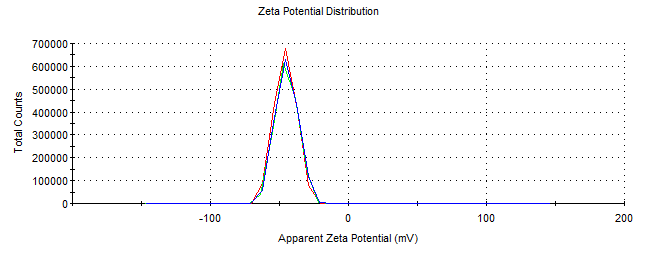


# b)

**S1 Fig.** Zeta potential of a) CNCs40 and b) CNCs80.

Supplement: S1 Fig — Zeta potential of a) CNCs40 and b) CNCs80. (DOCX) [file pone.0246794.s004.docx]
