# Supplementary material for: Valorization of khat (Catha edulis) waste for the production of cellulose fibers and nanocrystals
Source: PLoS One. 2021 Feb 9;16(2):e0246794. doi: 10.1371/journal.pone.0246794 (PMC7872298; doi:10.1371/journal.pone.0246794)
Supplement: S2 Fig — (DOCX) [file pone.0246794.s005.docx]

S2 Fig. Rheological profiles of diclofenac sodium gel formulations (F0-F5).
