# Supplementary material for: Valorization of khat (Catha edulis) waste for the production of cellulose fibers and nanocrystals
Source: PLoS One. 2021 Feb 9;16(2):e0246794. doi: 10.1371/journal.pone.0246794 (PMC7872298; doi:10.1371/journal.pone.0246794)
Supplement: S1 Table — (DOCX) [file pone.0246794.s006.docx]

S1 Table. Properties obtained from the (deconvoluted) XRD of untreated KW, as-obtained cellulose fibers, and CNCs.

| **Materials** | **d-spacings (nm)** | | | | **τ_200_ (nm)** | **X_200_** | **Δd/d_200_** | **CrI (%)** | **Z-Values** |
| --- | --- | --- | --- | --- | --- | --- | --- | --- | --- |
|  | **1**‐**10** | **110** | **200** | **040** |  |  |  |  |  |
| **KW-0** | 0.597 | 0.565 | 0.401 | 0.261 | 2.571 | 0.290 | 0.1528 | 55.29 | -47.91 |
| **C_40_** | 0.593 | 0.544 | 0.385 | 0.259 | 3.572 | 0.477 | 0.1006 | 73.61 | ‐35.74 |
| **C_80_** | 0.608 | 0.562 | 0.385 | 0.262 | 3.589 | 0.479 | 0.1003 | 78.00 | ‐26.58 |
| **CNCs_40_** | 0.589 | 0.528 | 0.394 | 0.260 | 5.466 | 0.630 | 0.0698 | 81.60 | ‐28.08 |
| **CNCs_80_** | 0.589 | 0.523 | 0.389 | 0.258 | 5.633 | 0.672 | 0.0681 | 82.84 | ‐23.57 |

(Key:- KW-0: untreated khat waste; C_40_ and C_80_: cellulose fibers obtained from khat waste with 40% formic acid and 40% acetic acid, and 80% formic acid and 80% acetic acid, respectively at the pretreatment stage; CNCs_40_ and CNCs_80_: cellulose nanocrystals isolated from C_40_ and C_80_, respectively; d-the interplanar spacing of the crystal; τ_200_‐average thickness of cellulose crystallites; X_200_‐the proportion of crystallite interior chains for the 200 plane; Δd/d_200_‐the fractional variation in the plane spacing for the 200 plane; CrI (SA)-crystallinity index following Segal et al. approach).
