# Supplementary material for: Valorization of khat (Catha edulis) waste for the production of cellulose fibers and nanocrystals
Source: PLoS One. 2021 Feb 9;16(2):e0246794. doi: 10.1371/journal.pone.0246794 (PMC7872298; doi:10.1371/journal.pone.0246794)
Supplement: S2 Table — (DOCX) [file pone.0246794.s007.docx]

**S2 Table.** Summary of thermal properties of untreated khat waste, obtained cellulose, and CNCs.

| **Material** | **ΔT (^o^C)** | **T_max_ (^o^C)** | **Weight loss (%)** | **Weight loss rate (%/^o^C)** | **T_10%_; T_50%_ (^o^C)** | **Residue at 550 ^o^C (%)** | **Residue at 700 ^o^C (%)** |
| --- | --- | --- | --- | --- | --- | --- | --- |
| **KW-0** | 29.72-126.03 | 68.64 | 6.38 | 0.2141 | 226; 340 | 34.56 | 31.40 |
|  | 189.32-372.39 | 320.12 | 48.40 | 1.1199 |  |  |  |
|  | 402.27-550.0 | -- | 6.94 | -- |  |  |  |
| **C_40_** | 29.72-121.09 | 58.07 | 5.52 | 0.1962 | 244; 336 | 19.28 | 16.73 |
|  | 189.47-396.59 | 339.86 | 68.55 | 2.2346 |  |  |  |
|  | 404.94-550.0 | 473.20 | 5.38 | 0.0742 |  |  |  |
| **C_80_** | 29.72-121.18 | 55.45 | 4.85 | 0.1851 | 241; 337 | 20.26 | 16.31 |
|  | 189.47-403.86 | 340.30 | 67.00 | 1.9019 |  |  |  |
|  | 410.50-550.0 | 460.35 | 6.82 | 0.1015 |  |  |  |
| **CNCs_40_** | 29.72-124.65 | 62.96 | 4.61 | 0.1386 | 167; 336 | 29.22 | 23.03 |
|  | 129.98-296.07 | 232.59 | 37.99 | 0.6726 |  |  |  |
|  | 300.84-470.81 | 355.71 | 21.60 | 0.3338 |  |  |  |
| **CNCs_80_** | 29.72-108.28 | 66.84 | 3.85 | 0.1284 | 163; 347 | 31.86 | 26.70 |
|  | 121.90-268.63 | 213.16 | 34.07 | 0.7744 |  |  |  |
|  | 274.41-431.60 | 360.41 | 21.72 | 0.3376 |  |  |  |

Key:- ΔT: Temperature change where main weight loss occurs; T_max_: Maximum degradation (maximum weight loss) temperature; T_10%_; T_50%_: Degradation temperatures where 10% and 50% weight losses occur; TGA-Thermogravimetric analysis; DTG: Differential thermogravimetry; KW-0: untreated khat waste; C_40_ and C_80_: cellulose fibers obtained from khat waste with 40% formic acid and 40% acetic acid, and 80% formic acid and 80% acetic acid, respectively at the pretreatment stage; CNCs_40_ and CNCs_80_: cellulose nanocrystals isolated from C_40_ and C_80_, respectively).
