# Supplementary material for: Valorization of khat (Catha edulis) waste for the production of cellulose fibers and nanocrystals
Source: PLoS One. 2021 Feb 9;16(2):e0246794. doi: 10.1371/journal.pone.0246794 (PMC7872298; doi:10.1371/journal.pone.0246794)
Supplement: S3 Table — (DOCX) [file pone.0246794.s008.docx]

S3 Table. *In vitro* diclofenac sodium release characteristics from the gel formulations and fitting models.

| **Formula** | **% cumulative drug released/ cm^2^** | **R^2^** | | | **K (mg cm^-2^ h^-1/2^)** | **(D, cm^2^ sec^-1^) x 10^-5^** |
| --- | --- | --- | --- | --- | --- | --- |
|  |  | **Zero-order** | **First-order** | **Higuchi** |  |  |
| **F0** | 58.96 ± 3.99 | 0.882 | 0.935 | 0.986 | 7.9 ± 0.045 | 0.71 ± 0.018 |
| **F1** | 59.02 ± 0.51 | 0.702 | 0.753 | 0.985 | 9.1 ± 0.021 | 0.75 ± 0.008 |
| **F2** | 55.46 ± 0.08 | 0.782 | 0.788 | 0.981 | 5.4 ± 0.017 | 0.25 ± 0.002 |
| **F3** | 62.92 ± 3.79 | 0.881 | 0.926 | 0.984 | 10.1 ± 0.025 | 0.98 ± 0.003 |
| **F4** | 56.87 ± 0.96 | 0.944 | 0.966 | 0.985 | 7.4 ± 0.029 | 0.64 ± 0.004 |
| **F5** | 47.05 ± 0.11 | 0.929 | 0.940 | 0.982 | 4.8 ± 0.031 | 0.19 ± 0.002 |

where, R^2^= correlation coefficient; K= apparent release rate; D= Diffusion coefficient. Data were presented as the mean ± SD (n=3).
